# An Efficacy Assessment of Phosphate Removal from Drainage Waters by Modified Reactive Material

**DOI:** 10.3390/ma13051190

**Published:** 2020-03-06

**Authors:** Agnieszka Grela, Michał Łach, Janusz Mikuła

**Affiliations:** 1 Faculty of Environmental and Power Engineering, Cracow University of Technology, 31-155 Kraków, Poland; 2 Faculty of Materials Engineering and Physics, Cracow University of Technology, Warszawska 24, 31-155 Kraków, Poland; mlach@pk.edu.pl (M.Ł.); jamikula@pk.edu.pl (J.M.)

**Keywords:** phosphates, drainage waters, zeolites, reactive material, modification

## Abstract

Phosphates may pose a threat to the aquatic ecosystem when there is a connection or a path between the soil and the aquatic ecosystem. Runoff and drainage ditches connect arable land with the waters of the receiver. Phosphates in the runoff and the ditches contribute to the negative phenomenon of surface water eutrophication. In order to prevent it, certain reactive materials are used which are capable of the selective removal of compounds by way of sorption or precipitation. Zeolites can be distinguished among the many reactive materials. Within the present analysis, the modification of a reactive material containing zeolites was carried out using calcium hydroxide solutions of different concentrations. A certain concentration of calcium hydroxide was created for use in further studies. In order to characterise the new material, an analysis was done of the chemical and mineral composition, as well as the porous texture and morphology. The efficacy of phosphate removal for its typical concentrations in drainage waters in Poland was confirmed by way of an experiment. Using a modified reactive material as an element of landscape structures may reduce the negative impact of phosphates on the quality of surface water.

## 1. Introduction

Excessive phosphate fertilisation leads to the accumulation of different forms of phosphorus in soil (including phosphates) [1] and contributes to the transportation of this element to the aquatic ecosystem [2]. Too high concentrations of phosphates in the aquatic system lead to eutrophication which negatively impacts recreation, the diversity of ecosystems and the treatment of drinking water [3]. The eutrophication of rivers is an equally common problem, as is the eutrophication of lakes, however the phenomenon present in rivers has not raised so much attention. This is caused by the fact that the effects of eutrophication (in the form of organic life transformation) in rivers are not as serious as in lakes.

Phosphorus in the form of phosphates reaches the river waters directly and/or via the riverine buffer zone from the drainage waters. The drainage waters come from the soils enriched in phosphates. This type of water, thanks to the effective transport pathways in the form of drains and drainage ditches, supplies—in a relatively short time—the river ecosystems [4]. In Poland, comprehensive research into the quality of this type of water was done by Professor Janusz Igras and his team [5]. New ways of reducing the transport of phosphates from arable lands to the aquatic ecosystem are constantly sought after. It still remains an open, serious and costly problem in all of the countries of the European Union [6,7]. In order to achieve the goal defined in the Water Framework Directive, namely good quality of water and good condition of the river ecosystems, it is necessary to reduce phosphate quantities from the arable lands in Europe and other countries [8,9]. Because of the dispersed quantities containing biogenic substances, agriculture introduces 45% of the total phosphorus quantity into the rivers. The negative impact of these areal sources of contamination affects the quality of over 40% of rivers and coastal waters [10] and, in the last half of the century, the phosphorus concentration has increased by 75% [11]. This justifies the subject matter of the present analysis, which is related to the removal of phosphates from drainage runoff in order to reduce their supply to the river surface waters.

The aquatic ecosystem can be contaminated by phosphates by local and areal agricultural sources [12]. Local contaminations are mainly related to the area of a farmstead. The size and scale of these contaminations depend on the water and sewage infrastructure within the farm, including tanks and panels for tipping off and storing natural fertilisers (solid and liquid), access to sewer systems or sewer treatment plants. Areal contaminations (discharged with water from arable land) are mainly associated with plant production. The size of these contaminations is difficult to locate or define as they depend on natural factors, e.g., climate and soil, as well as agricultural activity. The size of the areal contaminations is determined on an approximate basis, mainly by calculating unit runoff coefficients. The cause of areal contaminations is the incomplete utilisation of the components introduced during agricultural production to the field level [13]. The components not used by plants accumulate in soil or become dispersed into the water and groundwater environment. Thus, they pose a threat to the drainage water, which is the starting point of the path that nutrients move down within a catchment.

Drainage waters come from drains or drainage ditches. They mark the beginning of the migration path of mineral components within a catchment. They are mainly supplied by waters from atmospheric precipitation. Mineral components reach surface waters through drainage waters, then confined groundwaters, and also deep waters. A faster water cycle as a result of draining off a particular area may have a greater impact on the size of surface water contamination than increased fertilisation has. The quality of drainage waters may be an indication of the scale of areal contamination within the catchment.

In Poland, the State Environmental Monitoring system excludes these waters from its scope. Drainage waters have been researched by Professor Janusz Igras and his team under a separate project financed by the Ministry of Agriculture and Rural Development. Monitoring research projects were conducted in a network of selected control farmsteads. The examinations were done by the National Chemical and Agricultural Research Laboratory in Wesoła, as well as its local divisions. The Institute of Soil Science and Plant Cultivation—State Research Institute in Pulawy supervised these monitoring research activities in terms of methodology [5]. 

Research works were conducted at selected production fields (arable land) and permanent pastures in direct access to drainage waters. Drainage waters were collected each year at specific measuring points, typically from several fields within a given farmstead. Sample collection at the outlets of the drains and drainage ditches (at a distance from the outlets) were done during the spring and autumn periods. In the springtime, water was collected during approximately one month between the snow melting and the beginning of plant vegetation. In the autumntime, water was collected during a period from harvest until the beginning of works in the field. All samples were collected according to a uniform procedure, then secured and transferred for analyses done at the local divisions of the Chemical and Agricultural Research Laboratory.

Based on the conducted monitoring examinations and the obtained results, five classes of drainage waters quality were established. Table 1 illustrates the classification ranges of phosphate concentrations for two distinguished types of drainage waters (drains and drainage ditches).

The established drainage water classification was accepted based on the total average phosphate concentration from two sample collection dates. The phosphate concentrations were found to be different based on the analyses done on the waters from the drainage ditches and drains. This prompted the decision to distinguish separate quality classification for the drains and drainage ditch waters. Different phosphate concentrations correspond to the same quality classes of the drains and drainage ditch waters. The drainage water classifications differ from the quality norms applicable to the surface and underground water [5].

In order to reduce the negative impact of phosphates on the quality of surface water, there are three main solutions implemented. First, on a small scale, wetlands are used as their soil and plant characteristics allow for partial removal of phosporus compounds [14]. Second, utilising reactive filtering structures applied as buffers, selectively removing some of the phosphorus compounds from the discharge of the drains and drainage ditch waters [15]. Third is a so-called “dispersed solution” where the reactive material can be applied as a barrier located in a ditch or around the drainpipe [6,7,16,17,18].

The element combining all of these solutions is an effective material capable of selective phosphate removal. In their studies, numerous researchers have described results of testing different types of materials for phosphate removal [19,20,21]. It should be noted that the majority of these studies were conducted under conditions of high phosphate concentration present in sewage and characteristic for their process conditions [22,23,24]. In this situation, the obtained results cannot be directly transferred onto the assessment of phosphate removal from drainage waters in which phosphate concentrations are relatively low. This means it is necessary to further develop separate research in this respect, looking for suitable reactive materials—adequate for effective removal of phosphates from this type of waters. In order to address these problems, this study involved analyses of phosphate removal (using modified reactive material) for typical concentrations present in drainage waters in Poland.

## 2. Reactive Materials Used for Phosphate Removal

Reactive materials are capable of the selective removal of compounds by way of sorption or precipitation [19,25,26]. Reactive materials dedicated to removing phosphates are solids capable of removing phosphates from a solution [27]. These materials are rich in aluminium (Al), iron (Fe) or calcium (Ca) as these elements are most typically responsible for phosphate binding or precipitation [20,28,29,30,31]. Magnesium (Mg) can also, to some extent, bind phosphates; however, it is not as effective as Al, Fe and Ca. What is crucial is for the potential reactive material not to contain any compounds which may be rinsed out from the filter and, subsequently, reach the already purified waters as the device is in operation. Reactive materials are the main component of structures used for removing phosphates because they constitute a “filter” which allows for removing phosphates from the contaminated water while they remain within the structure of the reactive materials. Due to the fact that reactive materials are non-consolidated, they contain openings in the form of pores which allow water to go in and penetrate the material, enabling direct contact between the solids and the liquids containing phosphates [6,7,21]. Table 2 summarises some of the reactive materials used for removing phosphates of agricultural origin.

Since the 1960s, researchers around the world have been conducting studies on phosphate removal materials [31,77,78]. Due to their origin, reactive materials are often divided into three categories: natural materials, industrial products and waste materials [19]. Natural materials include apatite, bauxite, dolomite, calcite/aragonite (limestone), gravel, sand, clay, “opoka” rock, peat, wollastonite, zeolites and tree bark. Man-made industrial materials include lightweight aggregates (LWA) or lightweight expanded clay aggregate (LECA), Filtralite^®^–P (modified material LECA), iron oxides and concrete [6,19,20,21,78]. Waste materials include different forms of coal ash, fly ash, ochres, and slag. 

Reactive materials can also be classified according to their chemical composition: metals, mainly materials containing Fe/Al, materials containing soluble divalent salts of alkaline earth metals (Ca and Mg) or their mixtures [78].

### 2.1. Zeolites as Reactive Materials 

Zeolites are among several reactive materials mentioned in Table 2. The term was first used in 1756 by a Swedish mineralogist A. F. Cronstedt. While analysing a newly discovered mineral, the researcher observed that it loses water when heated. Cronstedt named the mineral zeolite, meaning “boiling rock” in Greek [79]. Since that time, zeolites have been considered a separate group of minerals [80]. 

Zeolites are defined as three-dimensional inorganic polymers formed from the SiO_4_ tetrahedra, which may be partially replaced by the tetrahedra of AlO_4_ [81]. Replacing some of the atoms of silicon Si^4+^ with aluminium Al^3+^ results in the excess of the negative charge of the crystal structure. In order to balance the charge, the presence of ion-exchangeable cations is necessary, for example, Na^+^, K^+^, Ca^2+^, to maintain the neutral charge of the whole framework. 

Zeolites are formed of three types of components: ion-exchangeable cations, crystal structure and zeolite water [82]. When heating up zeolites to 400 °C, this water is released in a continuous manner, not affecting the shape of the zeolite crystals. In humid environments, zeolites have the capability of absorbing water molecules through the crystal structure [83]. 

It is characteristic for zeolites’ crystal structures to contain empty spaces in the form of chambers and channels. Their sizes range between 3 Å to 30 Å. The specific internal structure of zeolites gives them various beneficial physical and chemical features which make them usable in industrial processes. The size of the channels within zeolites is large enough to enable not only individual atoms, but also the molecules of chemical compounds to diffuse and penetrate them [84].

Zeolites can be subdivided into natural and synthetic. Natural zeolites are formed by way of rock transformations under hydrothermal conditions. The most typically occurring zeolites are clinoptylolite, mordenite, phillipsite, chabazite. Zeolites can also be formed by way of a chemical synthesis. In 1948, as a result of a synthesis, Barrer created the first natural zeolite analogue [85]. Zeolite synthesis is a complex physico-chemical process, conducted mainly under hydrothermal conditions in alkaline environments [86]. Resources used for synthesis can be clay minerals, silica minerals, as well as coal combustion by-products (fly ashes). Synthesis of the zeolites created under laboratory conditions is much shorter than zeolite creation in natural conditions, which takes several thousand years [87]. In practice, synthetic zeolites are used more often than natural ones [80]. Typically, before they are used, natural zeolites must undergo costly structure modification treatment. Compared to natural zeolites, the synthetics are characterised by better texture parameters. In creating synthetic zeolites, one can manage the conditions of the synthesis process in a way that allows the acquisition of zeolite materials of a structure type that will be optimal for a given application. The process of synthesis involves additional costs; therefore, the substrates should be inexpensive minerals or waste materials.

### 2.2. Reactive Materials Modification

In order to modify reactive materials used for phosphate removal, the following metals are often used: Zr^4+^, La^3+^, Al^3+^ or Fe^3+^. Using these metals, however, may be toxic to aquatic life. Releasing these metals, especially Al^3 +^ and Fe^3 +^, under redox or acidic conditions may also lead to the worsening of water quality [88,89,90]. Additionally, utilising chemical reagents containing rare earth elements (REE), e.g., La^3+^ (in a commercially modified bentonite known as Phoslock) [89] is much more expensive compared to utilising Ca^2+^, Fe^3+^ and Al^3+^. The European Commission described REE and magnesium as critical resources in terms of their increasing prices and the difficulty in accessing them [91]. This is why it is crucial to develop other, more cost-effective, locally available and environmentally friendly methods of pre-treatment which involve reactive material modification, especially when these materials are to contain nutrients which can be further used in agriculture as fertilisers, free from the constraints associated with the modifying reagents. Releasing cations, exchangeable or found on the surface of reactive materials, such as K^+^, Na^+^, Ca^2+^ and Mg^2+^ does not affect the environment because they are considered to be non-toxic [92]. Examples of other modifications carried out by the authors using calcium compounds are presented in Table 3.

The arguments supporting the idea of using calcium hydroxide for modification are the lack of negative impact on the environment and the economic considerations. Calcium hydroxide is cheaper than, for example, magnesium salts [98] which are commonly used in modifying the materials for phosphate removal [99].

Considering the fact that reactive materials capable of effectively removing phosphates of low initial concentrations, characteristic of, for example, drainage runoff, are still being sought after, we decided to modify the synthetically obtained zeolite materials using calcium hydroxide. Removing phosphates from aqueous solutions with the application of reactive materials, such as zeolites, is limited by the negative charge of the crystallite surface. In order to increase chemical affinity of the zeolite materials to these compounds, it is necessary to modify their surface using calcium compounds. Examples are given in Table 3. Modification is necessary in order to change the surface charge from negative to positive, which facilitates the adsorption of phosphorus anions and/or precipitation or creation of insoluble compounds [100]. 

There are a number of examples of modifying natural zeolites using calcium hydroxide in the body of literature. We decided to check if such modification can be effective for synthetic zeolite materials.

## 3. Materials and Methods 

### 3.1. Assumptions for the Experiment Related to the Choice of Ca(OH)_2_ Concentration Used for Modification

For modification of the synthesised reactive material, the tuff of Filipowice (TF), created in a process of synthesis by the fusion method [101], a Ca(OH)_2_ solution of 0.10 M concentration was used. 0.25 M; 0.50 M [95]. Constant process conditions were ensured; the ratio of the solid’s mass (the reactive material) to the volume of Ca(OH)_2_ solution was 1:10, and the process temperature was 20 °C. In a beaker, a solution of Ca(OH)_2_ of appropriate molar concentration was prepared. Distilled water was added to the pre-measured quantity of Ca(OH)_2_. In order to make Ca(OH)_2_ into a solution, it was dissolved by mixing it in with water by means of a magnetic stirrer for 1 h. The prepared Ca(OH)_2_ solution was added to the pre-measured quantities of reactive material placed in flasks and shaken at 150 rpm for 24 h. The obtained suspension of modified reactive materials was centrifuged at 4500 rpm for 10 min. Then, the solution was poured to create the samples and topped up with distilled water. The samples were shaken at 150 rpm for 10 min. The content of each flask was poured into a plastic bottle fitted with a cap and topped up with 1 dm^3^ of distilled water. The bottles were screwed tight and shaken again at 150 rpm for 1 h. They were then put away for 15 min, after which the samples were filtered using a vacuum water pump and fine filter papers. Material samples remaining on the filter paper were dried at 105 °C, and then rinsed again using the same procedure described above. The rinsing was done until the pH of the solution was similar to the pH of the reactive materials prior to the modification with Ca(OH)_2_.

In order to establish the efficacy of reactive materials modification with the following Ca(OH)_2_ solutions: 0.10 M, 0.25 M, 0.50 M, an initial experiment of phosphate removal was carried out. The conditions of the experiment were decided on based on the data from the available literature:

- Dose: 5.0 [g/dm^3^], according to Table 4;
- Phosphate concentrations: approximately 1.0 and 7.0 [mg/dm^3^], according to Table 1.

Expecting changes in phosphate concentrations over time, while wanting to select the appropriate Ca(OH)_2_ concentration for the modification, it was assumed that phosphate concentrations will be determined after: 24, 48, 72, and 144 h [95] of the contact time of modified reactive materials with phosphate compounds.

Initial and final phosphate concentrations within the compound were analysed using a colorimetric method with ammonium molybdate and ascorbic acid [102,103] on a Hitachi U–1800 spectrophotometer (Hitachi, Tokyo, Japan), at a wavelength of 870 nm. The method, utilising ascorbic acid, involves a reaction of ammonium molybdate and antimonium tartaricum in acidic environment with orthophosphates, thus forming phosphomolybdic acid which is subsequently reduced with ascorbic acid to an intensely coloured molybdenum blue. The standard solution of potassium dihydrogen KH_2_PO_4_ by Merck was used as the benchmark. All designations for each sample were done three times (samples were collected from three bottles). 

In order to select the concentration which would finally be used in the reactive material modification, the impact of different concentrations on the efficacy of phosphate removal from the prepared solutions was assessed. The efficacy of phosphate removal was defined using Equation (1):

(1) $$Skt=(\frac{c_{0}-c_{t}}{c_{0}})\times 100\%$$

where:

*Skt* is efficacy of phosphate removal [%];

*c*_0_ is initial concentration [mg/dm^3^];

*c_t_* is concentration after contact time, where t = 24, 48, 72, 144 [mg/dm^3^].

**Figure 1:**
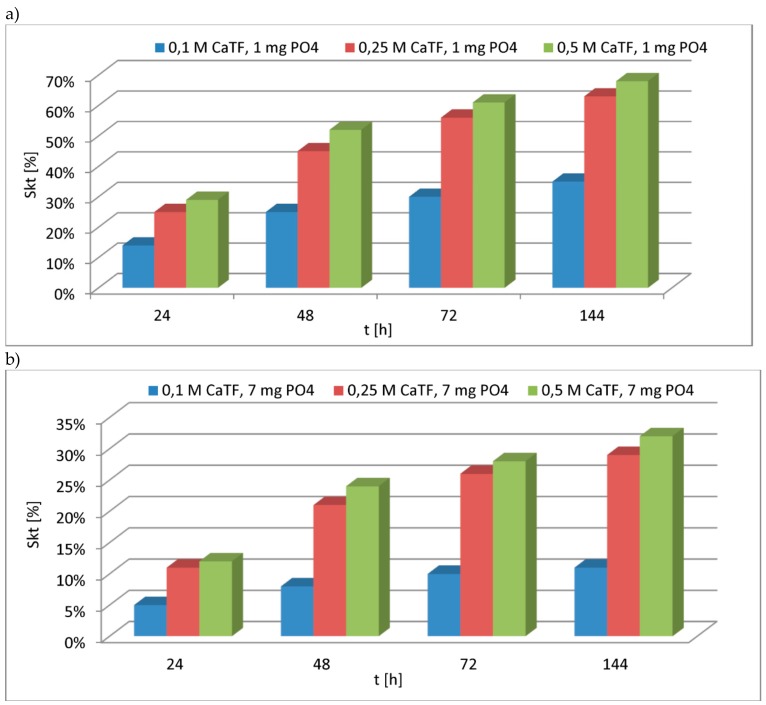
The percentage phosphate removal efficacy depending on the molar concentration of Ca(OH)_2_ used for the modification of a tuff of Filipowice (TF) sample. The provided results are an average of three designations. (**a**) Experiment parameters: Cp = 1.072 [mg/dm^3^]; dose = 5.0 [g/dm^3^]; time = 24–144 h; (**b**) experiment parameters: Cp = 7.067 [mg/dm^3^]; dose = 5.0 [g/dm^3^]; time = 24–144 h.

Two phosphate concentrations were used in the experiment (approximately 1.0 [mg/dm^3^] and approximately 7.0 [mg/dm^3^]), which corresponded to the characteristic concentrations of the drainage water (Table 1). After the set contact time elapsed, each of the modified materials’ phosphate concentrations were measured in the solution. Then, the efficacy of phosphate removal was calculated, and the results are presented in Figure 1a,b.

### 3.2. Determining The Chemical and Mineral Composition, As Well As The Porous Texture and Morphology of The Modified Reactive Material

Chemical composition was determined using X-ray fluorescence spectroscopy (WD–XRF) (PANalytical, Almelo, The Netherlands). A quality analysis of the spectrum was done by identifying spectral lines and determining their potential coincidences. Based on this, analytical lines were selected. A semi-quantitative analysis was designed using SQX calculation software (fundamental parameters approach). The analysis was done within the scope of fluoride–uranium (F–U), while the content of the determined elements was normalised to 100%. Standard preparation using the pastillation technique with the addition of a binder (Celleox) was applied. The ratio of sample to binder was 4.5:1.5.

For the analysis of the mineral composition, X-ray diffractometry (XRD) (PANalytical, Almelo, The Netherlands) was used. The designation of the phase composition was carried out by means of an X-ray, using the powder (Debye–Scherrer) method. X-ray images registered by means of the Rigaku SmartLab X-ray diffractometer, applying the Cu lamp, reflection graphite monochromator. The analysis was carried out within the angular scope 5–70° 2θ; the calculation time corresponding to one time step was 1 s. The obtained values of inter-surface distances were used for the identification of the phases contained in the tested samples, based on the data included in the International Centre for Diffraction Data (ICDD) 2014 catalogue and the XRAYAN software. 

The porous texture characteristics were determined based on the isotherms of low-temperature adsorption and desorption of nitrogen at 196 °C. The analysis was carried out using ASAP 2020 (Micromeritics) designed for precision sorption measurements. Prior to measuring, samples were vacuum heated at 150 °C for 12 h. The following parameters of porous texture were calculated for these samples:

1. Specific surface area (SSA_BET_) according to the methodology by Brunauer–Emmett–Teller;
2. Pore total volume V^0,99^_tot_ for relative pressure p/p0 = 0.99;
3. Micropore volume V^DR^_mik_ (pores of width smaller than 2 nm) according to the methodology by Dubinin–Radushkevich;
4. Mesopore volume V^BJH^_mez_ (pores of width greater than 2 nm and smaller than 50 nm) according to the methodology by Barrett–Joyner–Halenda (BJH).

Macropore volume was calculated based on the total difference between pore volume and the volume of micro- and mesopores. The analysis was conducted as recommended by the norms: ISO 9277:2010(E), ISO 15901–2:2006(E), ISO 15901–3:2007(E).

Morphology was analysed using a JEOL JSM–820 scanning microscope (Joel, Tokyo, Japan). Samples were previously adequately prepared. Small amounts of the materials were dried out to a solid mass and then placed on a carbon medium, ensuring charge dissipation from the sample. Materials were coated with a thin layer of gold using the JEOL JEE–4X evaporator (Joel, Tokyo, Japan).

### 3.3. The Experiment Methodology of Phosphate Removal Using Calcium Hydroxide Modified Reactive Material

The phosphate removal experiments were carried out in order to assess and compare the efficacy of phosphate removal using modified reactive material CaTF, i.e., the tuff of Filipowice created in a process of synthesis by the fusion method and modified by 0.25 M Ca(OH)_2_. 

The experiments were carried out using statistical method in a closed circuit [104]. This means that the known (and unchanged during the course of the experiment) volumes of solutions containing phosphates reacted with the reactive materials in a given time: 1, 4, 24, 48, 72, and 144 h [95]. Compared to the experiment done using dynamic method (a solution flows through a column containing the reactive material), the experiment done using the statistical method was characterised by a lack of flow or constant mixing of phosphate solutions in with the reactive material.

Samples of modified reactive material used in the experiment were dried out. Modified reactive material samples of the following quantities: 500, 1000 and 2000 ± 2.0 mg per sample were placed in plastic bottles. The volume of the bottles was 0.25 dm^3^. Individual doses of material were topped up with 0.20 dm^3^ of solution containing appropriate phosphate concentration. The assumed material doses correspond to the ratio of solid mass to the solution volume: 2.5, 5.0 and 10.0 [g/dm^3^]. After the analysis of the phosphate concentrations listed in Table 1, it was decided that the experiments testing the use of modified reactive materials for phosphate removal will use the following concentrations of these compounds: approximately 1.0, 3.2 and 7.0 [mg/dm^3^] which approximately corresponds to the phosphate concentrations found in the drainage waters of classes III–V [5]. 

Phosphate solutions were prepared by dissolving appropriate quantities of monopotassium phosphate (KH_2_PO_4_) in distilled water. The contents of bottles were mixed for 60 s and then screwed tightly with a cap in order to reduce evaporation. Phosphate concentrations in each bottle were determined at each of these time steps (t): 1, 4, 24, 48, 72, and 144 h. The collected samples were filtered using a 0.45 μm syringe filter. 

Phosphate concentrations measured at selected time steps (*c*_t_) were analysed using a colorimetric method with ammonium molybdate and ascorbic acid [102,103] on a Hitachi U–1800 spectrophotometer (Hitachi, Tokyo, Japan), at wavelength 870 nm. All designations for each sample were done three times (samples were collected from three bottles). 

Based on the literature review presented in Table 4, the following doses of modified reactive materials were selected: 2.5, 5.0 and 10.0 [g/dm^3^].

## 4. Results and Discussion

### 4.1. Reactive Materials Modification With Calcium Hydroxide

Comparing the efficacy of removing phosphates from a solution of initial concentrations at approximately 1.0 [mg/dm^3^] and 7.0 [mg/dm^3^] using the TF material modified with solutions of calcium hydroxide (0.10 M and 0.25 M), it can be observed that the efficacy is higher when modification is done using a solution of higher concentration (Figure 1a,b). The efficacy of phosphate removal from a solution of initial concentration of approximately 1.0 [mg/dm^3^] for all of the analysed contact time steps increased nearly two-fold (for instance, after 24 h: 14% for 0.1 M CaTF, 24% for 0.25 M CaTF). This effect (of improving phosphate removal efficacy) was insignificant when modification was done using 0.50 M solution (for instance, after 24 h: 24% for 0.25 M CaTF, 29% for 0.50 M CaTF). 

Analysing the results of phosphate removal efficacy from solutions of approximately 7.0 [mg/dm^3^] using 0.10 M CaTF and 0.25 M CaTF (Figure 1) materials, the improvement was over two-fold (for instance, after 48 h: 8% for 0.10 M CaTF, 21% for 0.25 M CaTF). Using the highest Ca(OH)_2_ concentration for modification improved the phosphate removal efficacy only slightly compared to the efficacy obtained when the 0.25 M solution was used (for instance, after 72 h: 26% for 0.25 M CaTF, 28% for 0.50 M CaTF). Considering that using the 0.5 M solution of Ca(OH)_2_ does not improve the phosphate removal efficacy in a considerable way, it was decided that, for further analyses, reactive materials will be modified using the 0.25 M solution of Ca(OH)_2_.

### 4.2. Modified Reactive Material Characteristics

Chemical analyses of the main elements converted into oxides presented in Table 5 proved that, in a sample containing the CaTF material, the dominant component is silicon, and—right after this—aluminium. Iron and magnesium occur in far smaller quantities. The calcium content was 15.72%. In the analysed sample of the CaTF material, low contents of sodium and potassium were found.

**Figure 2:**
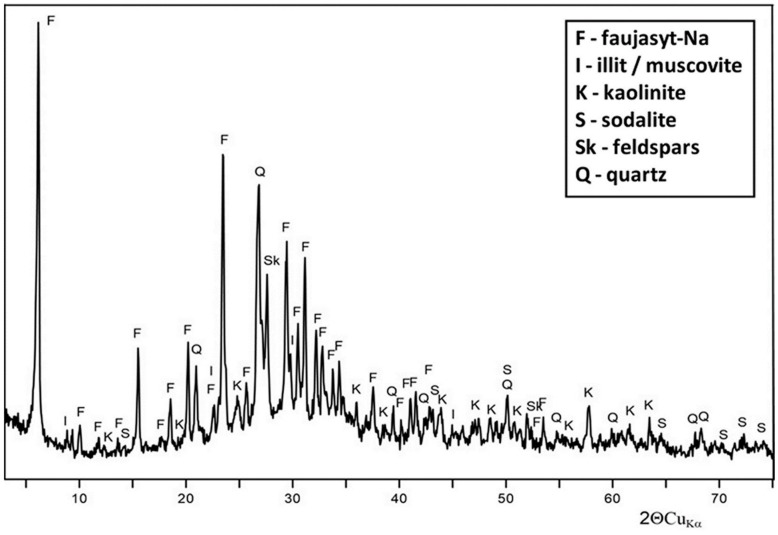
Diffractogram of the Filipowice tuff created in a process of synthesis by fusion method and modified with 0.25 M of Ca(OH)_2_ (CaTF).

Mineral composition of the modified reactive material CaTF was dominated by faujasite-Ca and illite/muscovite. Kaolinite and sodalite can also be found in smaller quantity (Figure 2). The XRD results presented in Figure 2 show a considerable background increase as a result of the increased amorphous phase.

The results of the porous texture tests done are presented in Table 6. After modification with 0.25 M of Ca(OH)_2_, the specific surface area of the CaTF material was 276.0 m2/g (CaTF). The modification caused an increase in the total pore volume compared to the material’s prior condition (TF) [101]. For the CaTF sample, the dominant type of porosity after the modification was mesoporous. Compared to the total calculated porosity volume, the share of mesopores was 55% (CaTF). For the CaTF material, the share of micropores was 36%. The percentage share of macropores also changed; the CaTF material was characterised by a 9% share of macropores out of the total porosity.

**Figure 3:**
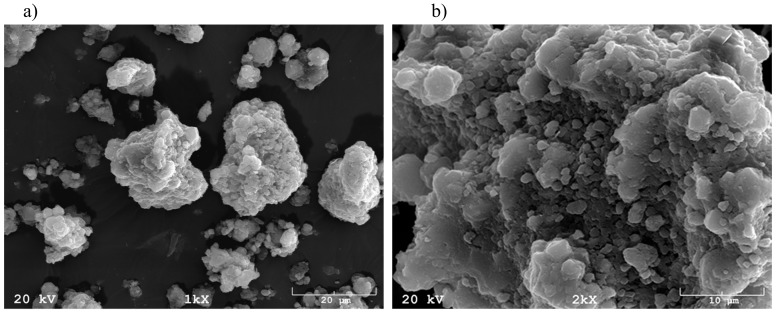
A SEM microscope image for a sample of the tuff of Filipowice created in a process of synthesis by fusion method and modified with Ca(OH)_2_ (CaTF); (**a**) zoom 1000 ×; (**b**) zoom 2000 ×.

In the images created using a scanning electron microscope (Figure 3a,b) it can be observed that the 0.25 M solution of Ca(OH)_2_ used for the modification reacted with the matrix constituted by the products which underwent a fusion method-based synthesis and created a dense layer on top of the crystal surface. This is reflected in the analyses of oxide content, in the form of increased CaO content in a material sample after the modification (Table 5; CaTF–15.72%). The crystals of the post-synthesis and post-Ca(OH)_2_ modification materials can function as a medium and, thanks to the macropores, they can contribute to phosphate precipitation [29].

### 4.3. The Efficacy of Phosphate Removal Using Modified Reactive Material

**Figure 4:**
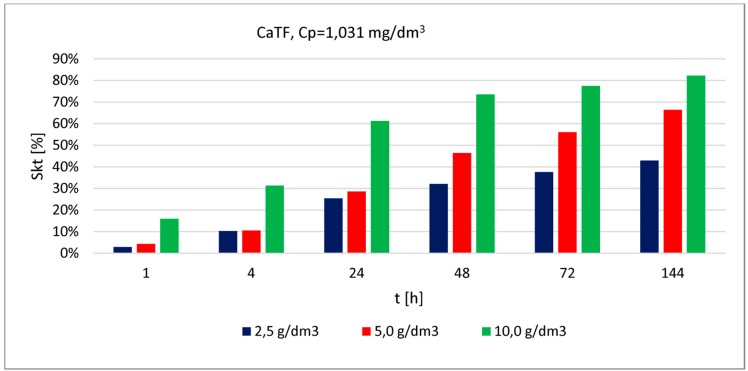
Phosphate removal efficacy (1.031 [mg/dm^3^]) by CaTF (dose: 2.5; 5.0; 10.0 [g/dm^3^]) at different contact time steps.

**Figure 5:**
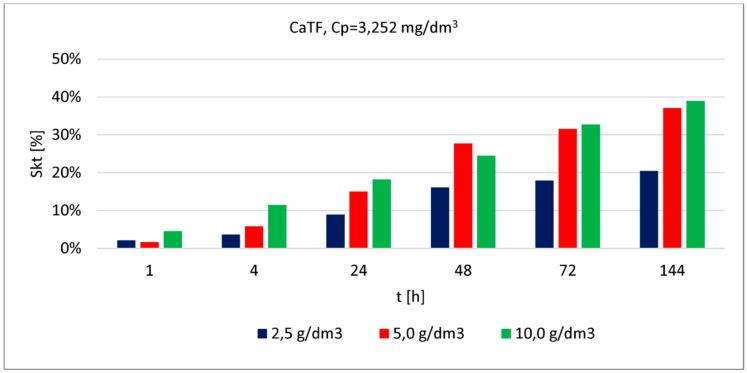
Phosphate removal efficacy (3.252 [mg/dm^3^]) by CaTF (dose: 2.5; 5.0; 10.0 [g/dm^3^]) at different contact time steps.

**Figure 6:**
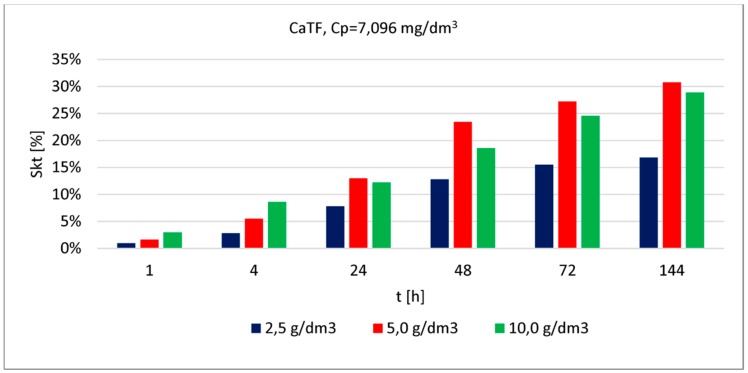
Phosphate removal efficacy (7.096 [mg/dm^3^]) by CaTF (dose: 2.5; 5.0; 10.0 [g/dm^3^]) at different contact time steps.

The phosphate removal efficacy was assessed based on a series of examinations carried out using modified reactive material (Figure 4, Figure 5 and Figure 6). In each of the graphs, the phosphate removal efficacy was presented depending on the three material doses used in the testing and different contact time steps between the material and solution.

Phosphate removal efficacy (for the lowest concentration) obtained by CaTF ranged between (depending on the applied material dose) 3% and 16% (after 1 h of contact) and 43% and 82% (after 144 h of contact). The highest removal efficacy values were observed for the highest dose (10.0 [g/dm^3^]), irrespective of the contact time or the initial concentration. It can also be observed that after 24 h, phosphate removal efficacy for the lowest concentration and doses 2.5 [g/dm^3^] and 5 [g/dm^3^] is similar, respectively 25% and 29%. Increasing the dose up to 10.0 [g/dm^3^] causes the removal efficacy to increase two-fold and reach 61%. A longer contact time, from 24 to 48 h for the 5.0 [g/dm^3^] dose causes the most considerable change in phosphate removal efficacy (by 17%). For higher concentrations (3.252 [mg/dm^3^] and 7.096 [mg/dm^3^]), even when applying the highest dose and the longest contact time, the resulting removal efficacy was at 39% and 29%. However, there is a clear removal efficacy improvement observed after extending the contact time from 24 to 48 h, respectively, by 13% (for the average concentration) and 10% for the highest. Assessing phosphate removal efficacy for the three analysed concentrations depending on the increasing doses of material can be presented in the following order: 1.031 [mg/dm^3^] > 3.252 [mg/dm^3^] > 7.096 [mg/dm^3^]. 

The testing results of phosphate removal efficacy (in terms of the concentrations similar to the ones analysed in this study), depending on the applied dose and different contact time steps, have been also described by other authors (Table 7).

Phosphate removal efficacy was tested using a natural zeolite from Thessaloniki in North Greece, modified using a 0.25 M solution of Ca(OH)_2_. The experiment was carried out for a phosphate solution of 10.0 [mg/dm^3^] concentration and a material dose of 10.0 [g/dm^3^], solution pH of 7.0 and the contact time of 120 h. According to the authors, the phosphate removal efficacy was 93.5% [95]. Other authors tested phosphate removal efficacy using autoclaved aerated concrete (AAC). Experiments were carried out for three doses of the reactive material: 1.0 [g/dm^3^]; 5.0 [g/dm^3^] and 10.0 [g/dm^3^], and a phosphate solution of concentration 2.972 [mg/dm^3^]. Phosphate concentrations were designated after different contact time steps. Reactive materials in the form of a filter were placed in the phosphate solution. The authors report that, after 24 h, the following removal efficacies were observed: 33% (for a 1.0 [g/dm^3^] dose), 56% for a 5.0 [g/dm^3^] dose) and 80% (for a 10.0 [g/dm^3^] dose). After two days, the phosphate removal efficacy increased up to 50% (for a 1.0 [g/dm^3^] dose), 80% for a 5.0 [g/dm^3^] dose) and 90% (for a 10.0 [g/dm^3^] dose). After six days, the phosphate concentrations measurements taken showed efficacy at 56% (for a 1.0 [g/dm^3^] dose), 83% for a 5.0 [g/dm^3^] dose) and 93% (for a 10.0 [g/dm^3^] dose). The authors report that after 30 days, the removal efficacies were, respectively, 88.5% (for a 1.0 [g/dm^3^] dose); 99.8% (for a 5.0 [g/dm^3^] dose) and 99.9% (for a 10.0 [g/dm^3^] dose) [105]. Other authors obtained similar results of phosphate removal efficacy for AAC after 8 h of sample shaking. Extending the contact time and sample shaking did not result in a better phosphate removal efficacy [63]. In other studies, estimating the efficacy of phosphate removal from solutions of 0.56 [mg/dm^3^] and 1.00 [mg/dm^3^] concentrations, the authors also used the AAC and observed a 95% removal efficacy only after 48 h, irrespectively of the initial concentration. The authors report that after only 10 h of contact time between the reactive material and the solution, the phosphate removal efficacy was at 70% [56]. The authors also used the lightweight fly ash aggregate Pollytag^®^. However, with concentrations ranging between 1.0 and 3.0 [mg/dm^3^], the resulting phosphate removal efficacy was between 1% and less than 2.5%. According to the authors, when phosphate concentrations were higher, at 10.0 [mg/dm^3^], the removal efficacy increased up to 34% [108]. Phosphate removal efficacy was tested by the authors using different reactive materials. The authors carried out quick tests which involved mixing 10.0 [g/dm^3^] or 50.0 [g/dm^3^] of reactive materials with phosphate solutions for 15 min, and subsequently establishing the concentration of the phosphates remaining in the solution after this time elapsed. The following results were obtained: white brick (dose = 10.0 [g/dm^3^], initial phosphate concentration = 1.2 [mg/dm^3^]) and removal efficacy at 6.8%; crushed seashells (dose = 10.0 [g/dm^3^], initial phosphate concentration = 10.0 [mg/dm^3^]) and removal efficacy at 20%; Philippines limestone (dose = 10.0 [g/dm^3^], initial phosphate concentration = 1.4 [mg/dm^3^]) and removal efficacy at 14.6%; Philippines limestone (dose = 10.0 [g/dm^3^], initial phosphate concentration = 5.6 [mg/dm^3^]) and removal efficacy at 7.6%; limestone (dose = 10.0 [g/dm^3^], initial phosphate concentration = 5.0 [mg/dm^3^]) and removal efficacy at 36.0%; Polonite^®^ (dose = 50.0 [g/dm^3^], initial phosphate concentration = 1.0 [mg/dm^3^]) and removal efficacy at 97.8%; Polonite^®^ (dose = 50.0 [g/dm^3^], initial phosphate concentration = 5.0 [mg/dm^3^]) and removal efficacy at 98.8% [43]. Phosphate removal efficacy was also examined by using 1.0 g/dm^3^ of “opoka” rock and FerroSorp^®^, with the phosphate concentration in solution at 2.0 [mg/dm^3^]. The results prove that “opoka” rock was more efficient within short contact time, i.e., 5 min. The analysed reactive material was characterised by a 95% phosphate removal efficacy. FerroSorp^®^, after 5 min of contact time, reached a 13% phosphate removal efficacy [109].

## 5. Conclusions

The changes constantly occurring in the environment as a consequence of the varying economic conditions in Poland make it necessary to gain knowledge continually on phosphorus infiltrating the waters and solutions for reducing this phenomenon; this necessity formed the basis for conducting the present research and opening a discussion related to this issue. The scope of research realised in the course of the experimental study and the obtained results presented in this work allow for an assessment of the efficacy of removing phosphates using the tested modified reactive materials (the tuff synthesised by way of the fusion method). This may invite further research development and open a wider discussion of the subject matter. 

The conducted modifications of the reactive materials allowed for the selection of a concentration of calcium hydroxide, which was finally used to modify the reactive material. It was decided that, in order to modify the reactive material, a 0.25 M solution of Ca(OH)_2_ would be used, considering the insignificant improvement of the removal efficacy if a 0.50 M solution of Ca(OH)_2_ was used. The presented results prove that the CaTF material is effective and capable of the selective removal of phosphates of agricultural origin. The concentrations of phosphates analysed in the study were the typical values occurring in drainage waters.

Comparing the phosphate removal efficacy using the CaTF material used under this study and a natural zeolite from Thessaloniki (also modified with a 0.25 M concentration of Ca(OH)_2_), a conclusion can be drawn that the CaTF material showed a lower efficacy of phosphate removal at a contact time of 144 h and a dose of 10.0 [g/dm^3^]. Moreover, the autoclaved aerated concrete was one of the materials which removed phosphates from aqueous solutions faster and with higher efficacy compared to the tested modified CaTF material. In contrast, analysing the efficacies presented in Table 7 for the lightweight ash fly aggregate Pollytag^®^, white brick and the Philippines limestone, it can be observed that it is the CaTF material which shows higher efficacy of phosphate removal.

The tested reactive material CaTF could be used as a component of landscape structures used for removing phosphates which constitutes a “filter”, allowing for the removal of phosphates from contaminated water while they remain within the structure of the reactive material.

## Figures and Tables

**Table 1:** Drainage waters quality classification ranges in Poland [5].

Component	Facility	Quality Classification				
		I	II	III	IV	V
PO_4_ [mg/dm^3^]	Drains	<0.1	0.1–0.2	0.3–0.4	0.5–3.3	>3.3
	Drainage ditches	<0.1	0.1–0.5	0.6–2.0	2.1–6.4	>6.4

**Table 2:** Examples of reactive materials used for removing phosphates.

Reactive Material	Main Component (Al, Ca, Fe, Mg)	Literature
Marls and sands	Al	[32,33]
Apatites	Ca	[34]
FGD gypsum		[35,36,37,38]
An oil shale		[39]
Crushed concrete		[40,41]
Crushed seashells/marls		[42,43]
Limestones		[43,44,45,46,47]
Wollastonite		[28,48]
Fe modified sand	Fe	[16,49,50]
Biotites	Al, Fe	[51]
Laterites		[52]
Metal chips and iron dust		[53]
Lightweight aggregate (LECA)	Al, Ca, Fe	[43,54,55,56]
Zeolites		[57,58,59]
Bauxite production waste	Al, Ca, Fe, Mg	[37,60]
Drinking water treatment waste		[35,36,37,61,62,63]
Fly ash		[22,35,36,37,38,41,60,64,65,66,67,68,69,70,71]
Slag		[64,65,66,67,68,72,73,74,75,76]

**Table 3:** Calcium compounds modification of reactive materials.

Material	Calcium Compound	Literature
bentonite	Ca(OH)_2_	[93]
	CaO	[94]
clinoptylolite	Ca(OH)_2_	[95]
montmorillonite	CaCl_2_·2H_2_O	[96]
NaP1–FA zeolite	CaCl_2_	[97]

**Table 4:** Selected publications based on which doses of modified reactive materials were selected and used in the experiments.

Dose [g/dm^3^]	Literature	Doses Tested
1.0	[105]	+
5.0		+
10.0		+
3.0	[106]	—
5.0	[107]	+
8.0	[104]	—
10.0	[95]	+

+: tested; —: not tested

**Table 5:** Chemical composition of sample CaTF.

Material	Oxide Composition/Mass%								
CaTF	SiO_2_	TiO_2_	Fe_2_O_3_	Al_2_O_3_	CaO	MgO	K_2_O	Na_2_O	Ig
	37.07	0.57	3.32	16.03	15.72	1.15	2.64	1.54	21.52

**Table 6:** Porous texture of a modified reactive material sample (CaTF).

Sample Name	SSA_BET_^1^ [m^2^/g]	V^0.99^_tot_^2^ [cm^3^/g]	V^DR^_mik_^3^ [cm^3^/g]	V^BJH^_mez_^4^ [cm^3^/g]
CaTF	276.0	0.292	0.105	0.162

^1^ SSA_BET_ specific surface area according to the methodology by Brunauer–Emmett–Teller, ^2^ V^0.99^_tot_ pore total volume for relative pressure p/p0 = 0.99, ^3^ VDRmik micropore volume according to the methodology by Dubinin–Radushkevich, ^4^ VBJHmez mesopore volume according to the methodology by Barrett–Joyner–Halenda.

**Table 7:** The efficacies of phosphate removal from aqueous solutions using reactive materials.

No.	Reactive Material	Initial Phosphate Concentration [mg/dm^3^]	Reactive Material Dose [g/dm^3^]	Contact Time [h]	Phosphate Removal Efficacy [%]	Literature
1	zeolite from Thessaloniki modified with 0.25 M solution of Ca(OH)_2_	10.00	10.0	120	93.5	[95]
2	autoclaved aerated concrete (AAC)	2.97	1.0	24	33.0	[105]
			5.0		56.0	
			10.0		80.0	
			1.0	48	50.0	
			5.0		80.0	
			10.0		90.0	
			1.0	144	56.0	
			5.0		83.0	
			10.0		93.0	
			1.0	720	88.5	
			5.0		99.8	
			10.0		99.9	
3	autoclaved aerated concrete (AAC)	0.56	-	10	70.0	[56]
		1.00		48	90.0	
4	lightweight fly ash aggregate Pollytag^®^	1.00–3.00	-	-	1.0–2.5	[108]
		10.00			34.0	
5	white brick	1.20	10.0	0.25	6.8	[109]
6	crushed seashells	10.00	10.0	0.25	20.0	
7	Philippines limestone	1.40	10.0	0.25	14.6	
		5.60	10.0	0.25	7.6	
8	limestone	5.00	10.0	0.25	36.0	
9	Polonite^®^	1.00	50.0	0.25	98.8	
10	“opoka” rock	2.00	1.0	5 min	95.0	
11	FerroSorp^®^	2.00	1.0	5 min	13.0	
12	CaTF	1.031	2.5	1	3.0	in article
			5.0		4.0	
			10.0		16.0	
			2.5	4	10.0	
			5.0		11.0	
			10.0		31.0	
			2.5	24	25.0	
			5.0		29.0	
			10.0		61.0	
			2.5	48	32.0	
			5.0		46.0	
			10.0		73.0	
			2.5	72	38.0	
			5.0		56.0	
			10.0		77.0	
			2.5	144	43.0	
			5.0		66.0	
			10.0		82.0	
	CaTF	3.252	2.5	1	2.0	in article
			5.0		2.0	
			10.0		5.0	
			2.5	4	4.0	
			5.0		6.0	
			10.0		11.0	
			2.5	24	9.0	
			5.0		15.0	
			10.0		18.0	
			2.5	48	16.0	
			5.0		28.0	
			10.0		24.0	
			2.5	72	18.0	
			5.0		32.0	
			10.0		33.0	
			2.5	144	20.0	
			5.0		37.0	
			10.0		39.0	
	CaTF	7.096	2.5	1	1.0	in article
			5.0		2.0	
			10.0		3.0	
			2.5	4	3.0	
			5.0		6.0	
			10.0		9.0	
			2.5	24	8.0	
			5.0		13.0	
			10.0		12.0	
			2.5	48	13.0	
			5.0		23.0	
			10.0		19.0	
			2.5	72	16.0	
			5.0		27.0	
			10.0		25.0	
			2.5	144	17.0	
			5.0		31.0	
			10.0		29.0	
